# Twelve weeks of a diet and exercise intervention alters the acute bone response to exercise in adolescent females with overweight/obesity

**DOI:** 10.3389/fphys.2022.1049604

**Published:** 2023-01-04

**Authors:** Nigel Kurgan, Lauren E. Skelly, Izabella A. Ludwa, Panagiota Klentrou, Andrea R. Josse

**Affiliations:** ^1^ Department of Kinesiology, Faculty of Applied Health Sciences, Brock University, St. Catharines, ON, Canada; ^2^ Centre for Bone and Muscle Health, Brock University, St. Catharines, ON, Canada; ^3^ School of Kinesiology and Health Science, Faculty of Health, York University, Toronto, ON, Canada

**Keywords:** exercise, dairy, adiposity, sclerostin, OC (osteocalcin), CTX (C-terminal telopeptide of type 1 collagen), OPG (osteoprotegerin), RANKL (receptor activator for nuclear factor k B ligand)

## Abstract

**Introduction:** Exercise and consumption of dairy foods have been shown to improve bone mineralization. However, little is known about the magnitude and timing of their synergistic effects on markers and regulators of bone metabolism in response to acute exercise in adolescent females with obesity, a population susceptible to altered bone metabolism and mineral properties. This study examined the influence of twelve weeks of exercise training and nutritional counselling on the bone biochemical marker response to acute exercise and whether higher dairy consumption could further influence the response.

**Methods:** Thirty adolescent females (14.3 ± 2.0 years) with overweight/obesity (OW/OB) completed a 12-week lifestyle modification intervention involving exercise training and nutritional counselling. Participants were randomized into two groups: higher dairy intake (RDa; 4 servings/day; n = 14) or low dairy intake (LDa; 0-2 servings/d; n = 16). Participants performed one bout of plyometric exercise (5 circuits; 120 jumps) both pre- and post-intervention. Blood samples were taken at rest, 5 min and 1 h post-exercise. Serum sclerostin, osteocalcin (OC), osteoprotegerin (OPG), receptor activator nuclear factor kappa B ligand (RANKL), and C-terminal telopeptide of type 1 collagen (βCTX) concentrations were measured.

**Results:** While there was an overall increase in sclerostin pre-intervention from pre to 5 min post-exercise (+11% p = 0.04), this response was significantly decreased post-intervention (−25%, p = 0.03) independent of dairy intake. The OPG:RANKL ratio was unresponsive to acute exercise pre-intervention but increased 1 h post-exercise (+2.6 AU; p < 0.001) post-intervention. Dairy intake did not further influence these absolute responses. However, after the 12-week intervention, the RDa group showed a decrease in the relative RANKL post-exercise response (−21.9%; p < 0.01), leading to a consistent increase in the relative OPG:RANKL ratio response, which was not the case in the LDa group. There was no influence of the intervention or dairy product intake on OC, OPG, or βCTX responses to acute exercise (p > 0.05).

**Conclusion:** A lifestyle modification intervention involving exercise training blunts the increase in sclerostin and can augment the increase in OPG:RANKL ratio to acute exercise in adolescent females with OW/OB, while dairy product consumption did not further influence these responses.

## 1 Introduction

During adolescence, there is a high rate of bone remodelling, characterized by bone resorption and subsequent formation. The balance of these two processes determines bone growth/development and the peak bone mass that one will achieve in their lifetime. Higher achievement of peak bone mass following adolescence is associated with a reduced risk of fragility fractures with age (Boreham and McKay, 2011; Gordon et al., 2017), highlighting the importance of the adolescent time period for the prevention of low bone mass-induced fractures (Hernandez et al., 2003). Further, understanding factors (e.g., nutrient intake) and stimuli (e.g., loading and unloading) that influence bone metabolism [i.e., increased muscle force/contractions predictably drive bone growth (Bass et al., 2005)] are critical when designing lifestyle modification strategies to augment bone accrual in youth.

It has been previously thought that individuals with overweight or obesity (OW/OB) have higher bone mass (Fassio et al., 2018), but this point has recently been challenged, as adolescents with OW/OB have a lower bone mineral density (BMD) and bone quality compared to normal weight females when body mass is controlled for (Janicka et al., 2007; Pollock et al., 2007; Sioen et al., 2016). Additionally, lifestyle modification strategies that aim only to reduce body mass (i.e., through caloric restriction) may put adolescent females at risk of not only losing lean mass but also blunting bone growth and negatively influencing bone mineral properties (Rourke et al., 2003; Kaulfers et al., 2011; Chaplais et al., 2020). Thus, designing lifestyle interventions for adolescents with OW/OB still undergoing linear growth that involves elements of weight management (not necessarily weight loss), including healthy eating and exercise, are critical for not only improving body composition and associated cardiometabolic risk but also for optimizing bone mass accrual (Rajjo et al., 2017).

Dairy products have been shown to increase bone accrual in adolescent youth (de Lamas et al., 2019; Wallace et al., 2021), a finding repetitively confirmed by whole-body BMD measures (Chan et al., 1995; Cadogan et al., 1997; Du et al., 2004; Lau et al., 2004) or specific regions [pelvis (Lau et al., 2004), trochanter (Merrilees et al., 2000), and tibia (Vogel et al., 2017)], and act synergistically with exercise training to improve whole-body BMD compared to each alone (Gómez et al., 2021). We recently conducted a randomized controlled trial in adolescent females with OW/OB that involved a 12-week lifestyle modification intervention consisting of nutritional counselling and thrice-weekly supervised exercise training (Calleja et al., 2020). Those that were given 4 servings/d of dairy products [i.e., the recommended dairy group (RDa)] had an increased daily intake of bone-supporting nutrients, including protein, vitamin D, calcium, phosphorous, and potassium, while those that maintained a low dairy intake [0–2 servings/d; i.e., the low dairy group (LDa)] did not. The RDa group also had reductions in fat mass and increased lean mass that were both greater than the LDa group (Calleja et al., 2020), suggesting a beneficial effect of increased dairy intake for body composition. Following the intervention, the RDa group had reductions in resting levels of the bone resorption marker C-terminal telopeptide of type 1 collagen (βCTX) compared to the LDa group, which is likely related to the increased intake of nutrients that support bone growth found in dairy products (Josse et al., 2020).

Acute exercise typically leads to a transient increase in markers of bone resorption (e.g., βCTX) and upstream catabolic osteokines (e.g., sclerostin), with generally a lack of change in markers of bone formation [e.g., N-terminal propeptide of type 1 procollagen (P1NP)] and bone turnover [i.e., osteocalcin (OC)], as well as upstream anabolic osteokines [i.e., osteoprotegerin (OPG)]. This temporal response in bone biochemical markers to acute exercise is thought to be essential for improving bone mineral properties by initiating resorption of old damaged bone and a subsequent, slightly delayed (i.e., not immediate), replacement with new osteoid/bone (Dolan et al., 2020; Dolan et al., 2022). Therefore, alterations in the regulation of this process may prevent bone mass accrual. Indeed, the acute responses of some bone biochemical markers have also been shown to be negatively influenced by adiposity (Kurgan et al., 2020). While our lab found no difference in the βCTX response to acute exercise between adolescent females with normal weight and OW/OB, we did observe a larger and more sustained increase in sclerostin in adolescent females with OW/OB (Kurgan et al., 2020). This post-exercise increase in sclerostin, which was only seen in adolescents with OW/OB, is similar to what is typically seen in adults. Concomitantly, this study also demonstrated that adolescents with OW/OB had consistently lower OC compared to adolescents with normal weight (Kurgan et al., 2020), possibly indicating lower turnover rates (Dolan et al., 2020; Dolan et al., 2022). Indeed, previous studies report that obesity in adolescence may accelerate bone maturity at the expense of peak bone mass (De Leonibus et al., 2014; de Groot et al., 2017) and may even oppose the long-term bone benefits observed with exercise training in adolescents. Recent research has also demonstrated that long-term exercise training can blunt the acute exercise transcriptional program in muscle in normal weight adults (Norrbom et al., 2022). Similar adaptations may occur with bone. In contrast, exercise training may augment the bone response to acute exercise in adolescents with OW/OB given that they appear to have lower bone quality than adolescents with normal weight (Janicka et al., 2007; Dimitri et al., 2010). Thus, using a sub-group of participants from our main study (Calleja et al., 2020; Josse et al., 2020; Skelly et al., 2021), the present study aimed to determine whether the response of bone markers and osteokines, including βCTX, sclerostin, OC, OPG, and receptor activator of nuclear factor kappa B Ligand (RANKL), to acute exercise is altered after a 12-week lifestyle intervention involving nutrition counselling and exercise training in adolescent females with OW/OB, and whether increased dairy consumption could further influence the response. Understanding the impact of exercise training and dietary considerations on the short-term regulation of bone metabolism to an acute bout of exercise will provide insight into long-term adaptations to bone growth and development.

## 2 Materials and methods

### 2.1 Participants

This study represents a secondary analysis that answered a novel question from a previously published intervention (Calleja et al., 2020; Josse et al., 2020; Skelly et al., 2021). The present study includes the available blood samples from 30 (mean age 14.3 ± 2.0 years; range 10–18 years) out of 63 adolescent females who took part in a lifestyle modification, weight management parallel randomized controlled intervention trial entitled, “*Improving Diet, Exercise And Lifestyle (IDEAL) for Adolescents*,” which was registered at ClinicalTrials.gov (NCT02581813), and was approved by our institution’s Research Ethics Board (BREB file # 14-284). The *IDEAL for Adolescents* Study was a 12-week, diet and exercise intervention study in adolescent girls with OW/OB carried out in different waves from June 2016−October 2018. The primary purpose of the *IDEAL for Adolescents* Study was to assess the effect of consuming the recommended 4 servings/day of dairy products vs. a low dairy product diet (0–2 servings/day), along with mixed-mode exercise training as part of a weight management intervention, on body composition in female adolescents (aged 10–18 years) with OW/OB. The effects of the intervention on fasted, resting concentrations of selected markers of bone remodelling before and after the intervention have been published (Josse et al., 2020); however, the analyses presented herein on potential changes in the acute post-exercise response of bone markers and osteokines has not been previously published.

The *IDEAL for Adolescents* Study participants were recruited from the Niagara Region, Ontario, Canada. Eligibility required participants to be menarcheal, between the ages of 10 and 18 years, overweight (OW; ≥85–96 percentile BMI) or obese (OB; ≥97 percentile BMI) based on World Health Organization growth charts, low dairy consumers (0–2 servings per day), minimally active (activity 0–2 times per week), and otherwise healthy. Participants were excluded if they reported an allergy to dairy foods or lactose intolerance, were taking medications related to a chronic condition or one that affected bone health or were consuming vitamin or mineral supplements not prescribed by a physician. All eligible participants and their parents/guardians were invited to Brock University, where they provided informed assent and consent, respectively. The main CONSORT flow diagram has been previously published (Josse et al., 2020).

On their first visit to the lab, participants completed a general health questionnaire to document medical history and medication use. After all entry criteria were met, participants were stratified by BMI percentile (either OW/OB) and were randomly assigned (using a random number generator) to one of three different groups: recommended dairy (RDa), low dairy (LDa), or a no-intervention control using an unblocked random allocation ratio of 2:2:1. The no-intervention control group was not included in this study due to low sample size for the specific outcomes of interest (i.e., the bone markers’ responses to acute exercise).

### 2.2 Study design and procedures

Details on the design and procedures of the *IDEAL for Adolescents* Study were previously published in Calleja et al. (2020). Briefly, before commencing the intervention, participants visited the laboratory for baseline testing. Tests included anthropometric measurements and the first set (i.e., baseline) of blood samples taken at rest and in response to an acute exercise bout (described in detail in the following section). Participants were also instructed on how to properly complete a 7-day food record, which was used to examine their habitual diet/nutrient intakes. After the initial visit, participants returned to the laboratory for an exercise introduction session, where the parent/guardian and participant met their personal trainer, reviewed the exercise program, and outlined the exercise schedule for the next 12 weeks. After the exercise introduction session, participants had their first diet consultation with a registered dietitian who reviewed the baseline 7-day food record with them and their parent/guardian and gave instructions for beginning the dietary protocol. Participants in the RDa group were then provided with dairy products and general guidance regarding their consumption during this visit and every 2 weeks for the 12-week intervention.

### 2.3 Acute exercise session and blood sampling

The acute exercise bout was meant to examine the bone response to exercise; thus, we utilized a protocol that involves high-impact, weight-bearing loads, as we have done previously in adolescents (Falk et al., 2016; Dekker et al., 2017; Klentrou et al., 2018; Kurgan et al., 2020). Participants began the exercise session with a warm-up that included 5 min of low-intensity cycling (40 W of resistance) on a cycle ergometer. Once the warm-up was completed, participants were given a comprehensive explanation and demonstration of each of the circuits. The protocol included 120 jumps organized into five circuit training stations, which included box jumps (jump height was set at 25 cm), lunge jumps, tuck jumps, single-leg hopping and jumping jacks (MacKelvie et al., 2003). Each station included three sets of eight repetitions and participants were allowed 3 min of recovery between sets. In addition to the comprehensive explanation before beginning the exercise session, before beginning each station, participants were shown how to perform each exercise and familiarized themselves with each circuit type to ensure each jump was done with the correct technique during the trial to avoid injury, and promote full effort for consistency across trials. Each plyometric testing protocol was carried out by the same two researchers to ensure safety, technique, and effort were monitored.

Blood sampling occurred in the morning hours (between 0800 and 1100 h) to minimize any circadian rhythm variation in the serum biomarkers. Participants were asked to come to the laboratory fasted (∼10–12 h) and to avoid any vigorous or high-impact exercise for at least 24 h before testing. Upon arrival to the laboratory, if requested, a topical anesthetic cream, Emla (25 mg/g lidocaine +25 mg/g prilocaine), was applied to the antecubital fossa of the participant’s arm before blood sampling. Subsequently, height, weight and body composition were measured. Participants then sat down and rested for 10 min. This was followed by a rested, pre-exercise, fasted venous blood sample, which was drawn by a phlebotomist using a standard venipuncture procedure with a 23G butterfly needle and vacutainers [serum separator tubes (SST)] (cat#: 367983-1, BD, Mississauga, ON). Vacutainer tubes sat to clot (∼20 min) before being centrifuged at 1400 RCF (xg) for 15 min at 4°C. Serum was separated and aliquoted into 1.5 ml polyethylene cryotubes that were stored at −80°C for analysis upon study completion. Following the resting blood sample, participants were provided with a standardized light breakfast (one small granola bar, one banana, and water). The breakfast was provided to ensure participants had adequate energy during the exercise session. It was also low in protein and calcium to not allow for the acute intake of these nutrients to influence the bone turnover response to acute exercise (Scott et al., 2012; Shams-White et al., 2017; Bhattoa, 2018). Within 20 min, they began the 30 min plyometric exercise protocol followed by two more blood samples at 5 min and 1 h post-exercise.

### 2.4 Exercise intervention

Participants in both groups (RDa and LDa) completed a structured, supervised exercise training program over the 12 weeks (×3/week). The exercise intervention was individualized and based on the principles of progressive loading whereby the exercise trainers would change exercise variables (i.e., load/reps/duration/speed) to maintain a constant exercise stimulus. Each session lasted between 60–90 min and began with a plyometric-based (jumping) warm-up for 5–10 min, followed by 20–30 min of aerobic training (on either a treadmill, cycle ergometer, elliptical or rowing ergometer) and either 20–30 min of resistance training using free weights and machines (2× per week), or plyometric exercises (1× per week). Upon completion, participants cooled down by stretching and walking. Participants consumed a drink immediately after each training session. The RDa group drank one cup (250 ml) of 1% chocolate milk, and the LDa group drank one cup of a non-dairy, vitamin D- and calcium-free, carbohydrate-based, electrolyte drink. On the days participants did not receive formal exercise training, they were encouraged to increase their physical activity to achieve a predetermined number of steps during their leisure time.

### 2.5 Dietary intervention

Dietary counselling by a registered dietitian was provided five times during the study (weeks 0, 2, 4, 8, 12) to each participant and their parent/guardian, individually. Energy requirements/expenditures were calculated for each participant using predictive equations from the Institute of Medicine for girls with OW/OB (Institute Of Medicine Food And Nutrition Board, 2005). This was used to prescribe a diet for weight maintenance (and not weight loss) based on the participant’s age, height, and body mass. Participants were provided with an eating plan outlining how many servings from each food group they should consume [according to Canada’s 2007 Food Guide (Health Canada, 2007)]. All participants were counselled on consuming a healthy diet of fruit, vegetables, high fibre foods, whole grains, lean meats, and meat alternatives. Participants were also asked to avoid processed foods, foods high in “bad” fats (trans and some sources of saturated fat), sugar-sweetened beverages, pastries, and confections, and they were instructed not to take any vitamin or mineral supplements or fortified juices/drinks during the study.

The study was designed such that the RDa and LDa groups differed primarily in the source of protein they consumed, as the RDa group consumed half of their daily protein (∼20% of total energy intake) from dairy sources. Specifically, for the duration of the intervention (12 weeks), the RDa group was provided with 4 servings/day of mixed dairy products including two cups of 1% milk (white and chocolate), 2 × 100 g cartons of 0% or 2% MF Greek yogurt (any flavour) and 42 g of full-fat cheddar or marble cheese. The LDa group maintained their low dairy intake of 0–2 servings/day and continued to consume protein from other sources including meat, egg, fish, chicken, legumes, and grains. They were also asked to refrain from consuming calcium-fortified beverages/foods. Thus, as per the study design (i.e., due to the provision of dairy foods), the RDa group should consume greater levels of bone-supporting nutrients, namely protein, vitamin D, potassium, phosphorus, magnesium, and calcium than the LDa group. Of note, the intakes of some of these nutrients were already higher in RDa compared to LDa at pre-intervention (Table 1). Energy intake was also higher in RDa which likely contributed to the greater micronutrient intakes at baseline.

**TABLE 1:** Age, anthropometric, body composition, and nutrient intake at pre- and post-intervention for the higher dairy intake (RDa) and the low dairy intake (LDa) groups.

Variable	RDa (*n* = 14)			LDa (*n* = 16)			*p*-values		
	Pre-Intervention	Post-Intervention	Δ	Pre-Intervention	Post-Intervention	Δ	Group	Intervention	Interaction
Age (years)	13.8 ± 1.4	—	—	14.5 ± 2.0	—	—	0.3	—	—
Age from PHV (years)	2.0 ± 0.9	2.2 ± 0.9	0.2	2.3 ± 1.0	2.4 ± 1.0	0.1	0.5	<0.001	0.6
Height (cm)	165.7 ± 6.2	166.3 ± 6.3	0.6	164.1 ± 6.3	164.6 ± 6.3	0.5	0.5	0.002	0.9
Body mass (kg)	78.4 ± 13.9	77.8 ± 11.7	−0.6	78.0 ± 13.7	77.9 ± 14.2	−0.1	>0.9	0.5	0.7
Fat Mass (kg)	29.4 ± 7.4	27.9 ± 6.1	−1.5	28.7 ± 7.3	27.8 ± 7.8	−0.9	0.9	0.004	0.5
Energy Intake (kcal)	1,729 ± 393	1,755 ± 253	26	1,575 ± 400	1,410 ± 239	−165	0.01	0.4	0.2
Protein intake (g·kg bm^−1^·d^−1^)	0.90 ± 0.3	1.18 ± 0.3*^,#^	0.28	0.84 ± 0.3	0.90 ± 0.2	0.06	0.1	<0.001	0.008
Carbohydrate intake (g·kg bm^−1^·d^−1^)	2.85 ± 0.9	2.72 ± 0.8	−0.13	2.57 ± 0.9	2.25 ± 0.7	−0.32	0.2	0.1	0.5
Fat intake (g·kg bm^−1^·d^−1^)	0.92 ± 0.3	0.88 ± 0.2	−0.04	0.85 ± 0.4	0.74 ± 0.3	−0.11	0.3	0.2	0.5
Vitamin D (µg·d^−1^)	2.99 ± 1.4^#^	5.34 ± 1.2*^,#^	2.35	1.69 ± 1.1	1.67 ± 1.1	−0.02	<0.001	<0.001	<0.001
Calcium (mg·d^−1^)	794 ± 271^#^	1,306 ± 165*^,#^	512	513 ± 240	448 ± 162	−65	<0.001	<0.001	<0.001
Phosphorous (mg·d^−1^)	908 ± 368	1,477 ± 216*^,#^	569	771 ± 193	860 ± 174	89	<0.001	<0.001	<0.001
Potassium (mg·d^−1^)	1,697 ± 673^#^	2,413 ± 554*^,#^	716	1,588 ± 477	1,778 ± 402	190	0.03	<0.001	0.02

Data are reported as mean ± SD. Δ = post-intervention—pre-intervention in their respective units. A two-way RMANOVA, was used to examine main effects for group, intervention, and their “interaction” = group*intervention. *Bonferroni* correction was used for pairwise comparisons; * = *p*< 0.001 compared to pre-intervention for RDa, # = *p*< 0.05 for RDa, compared to LDa, at pre- or post-intervention; ANCOVA, was used for fat mass with change in body weight as the covariate.

### 2.6 Adherence

Adherence was calculated separately for the exercise and dairy components of the study as detailed in (Calleja et al., 2020). Briefly, adherence to the exercise training component of the intervention was calculated by comparing the number of exercise sessions attended to the number of sessions that were scheduled and converting it to a percentage. Adherence to the consumption of the dairy products (or not) was based on self-reported average daily servings of dairy products consumed by the participants at weeks 4, 8, and 12. Consumption was monitored and verified by the dietitian using specific daily checklists. RDa participants were considered adherent if they consumed ≥3 of the 4 prescribed servings/day. LDa participants were considered adherent if dairy servings intake was ≤2/day.

### 2.7 Anthropometrics, body composition and maturity

Height (cm), seated height (cm), body mass (kg), and body composition (lean mass and fat mass) were assessed for each participant at weeks 0 and 12 by the same investigator. Standing and seated height were measured using a stadiometer (Seca 213 Portable Stadiometer, CME Corp., Warwick, RI) to the nearest 0.1 cm with light clothing and no shoes. Body mass was assessed using a standard scale (Digital Physician Scale, Rice Lake Weighing Systems, Rice Lake, WI). Body composition was measured as previously described (Calleja et al., 2020), using the BodyMetrix (BMX; BodyMetrix System, BX-2000, IntelaMetrix, Inc., Livermore, CA), a handheld device that utilizes amplitude-mode ultrasound technology to measure fat thickness. The somatic maturity offset (years from peak height velocity) was estimated using a sex-specific regression equation (Mirwald et al., 2002). This is a simple, non-invasive method of assessing somatic maturity in children using known differential growth measures of height, seated height, and leg length.

### 2.8 Food records

Participants provided 7-day food records at weeks 0 and 12 and 3-day food records at weeks 2, 4, and 8 before each dietetic counselling session to assess dietary intake, to track compliance with the nutrition protocol, and to provide guidance moving forward. Food records were analyzed using the Food Processor Diet analysis software program (ESHA Research, Inc. Salem, OR).

### 2.9 Serum bone biochemical markers

The βCTX (β-CrossLaps; cat#: 11972308 122) was measured from serum at the Mount Sinai Hospital Core Laboratory (Toronto, Ontario) using a Roche Cobas e602 automated analyzer. Lower and upper detection limits were 0.010–6.00 ng/ml (quality control standard CV: 4.8%). Serum concentrations of sclerostin, OC, OPG and RANKL were measured in duplicate with intra-assay and inter-assay coefficients of variation (CVs) measured in house. Sclerostin was measured using an enzyme-linked immunosorbent assay (ELISA; cat# DSST00; R&D, Minneapolis, MN). OC and OPG were measured using a microbead multiplex kit (cat# HBNMAG-51K-08, EMD Millipore, Darmstadt, Germany) and RANKL was measured using a microbead single-plex kit (cat# HRNKLMAG-51K-01, EMD Millipore, Darmstadt, German). The average intra-assay CVs for sclerostin, OC, OPG, and RANKL were 5.4, 5.6, 4.8, and 6.1%, respectively. The average inter-assay CVs for sclerostin, OC, OPG, and RANKL were 5.7, 6.4, 5.1, and 4.1%, respectively.

### 2.10 Statistical analysis

Results are presented as mean ± standard deviation (SD) for all Tables and Figures. For age, anthropometrics, and nutrient/energy intake variables, two-way repeated-measures ANOVAs (RMANOVA) were used to examine main effects for group (differences between RDa and LDa) and intervention (pre- and post-intervention response), as well as their interaction (group*intervention) to examine if the groups responded differently to the intervention. In case of a significant interaction, a *Bonferroni* correction was applied accounting for the four *post hoc* pairwise comparisons. For fat mass, a 2-way repeated measures ANCOVA was used with weight change as a covariate as we have previously done (Calleja et al., 2020). Biochemical data were assessed for normality using skewness and kurtosis. For absolute concentrations of biochemical markers, sclerostin, βCTX, and OC were normally distributed, while RANKL and OPG:RANKL were not, but normality improved following log transformation. Of a total of 174–180 samples, missing datapoints (*n* = 3) were replaced with the group, intervention, and timepoint-specific mean value. In addition, individual outlying datapoints were identified (>±3 SD) and replaced with the corresponding group, intervention, and timepoint-specific upper or lower 3 SD limit, which resulted in 0/180, 4/180, 0/180, 11/174, 4/174, and 0/180 replaced datapoints (174–180 total datapoints in the dataset) for sclerostin, OC, OPG, RANKL, OPG:RANKL, and βCTX concentration, respectively.

Three-way RMANOVAs were performed to examine main effects for group (differences between RDa and LDa), intervention (pre- and post-intervention response), and the time response of bone biochemical markers (absolute concentrations and relative change from pre-exercise) to acute exercise and their interactions. For main effects in the three-way RMANOVA, short-term response to the acute exercise bouts was the main effect of time, comparison between pre- and post-intervention was the main effect of intervention, and the difference between groups (RDa vs. LDa) was the main effect of group. We also examined their interactions, which included time*intervention, time*group, intervention*group, and time*intervention*group. Following significant main effects or interactions, pairwise comparisons using a *Least Significant Difference (LSD)* correction were assessed. For all statistical tests, significance was assumed at an alpha level of <0.05. Sphericity was assumed at *p* > 0.05, and if <0.05, the Greenhouse-Geisser correction was used. Analyses were performed using SPSS version 26.0 for Windows (SPSS, Chicago, Illinois, United States) and graphs were made in GraphPad Prism 9 (San Diego, CA, United States).

## 3 Results

Adherence to the scheduled exercise sessions of the RDa group was 86% ± 9% and the LDa group 79% ± 11%. All (100%) of the RDa participants reported consuming ≥3 servings/day of dairy products and 100% of the LDa participants reported consuming ≤ 2 servings/day of dairy products. Specifically, the combined dietary intake data from weeks 4, 8 and 12, showed RDa reported 3.8 ± 0.4 servings/day and LDa reported 0.3 ± 0.3 servings/day.

Table 1 presents pre- and post-intervention data for demographic, anthropometric, body composition, and nutrient intakes between LDa and RDa groups. There was a significant main effect for group in energy intake with no intervention effect or interaction, which was a result of the RDa group having higher energy intake compared to the LDa group both at pre-intervention (mean difference = +249 kcal) and post-intervention (mean difference = +344.8). While there was no difference between groups at either pre- or post-intervention in carbohydrate and fat intake, there was a group*intervention interaction for protein intake. This was due to RDa increasing their protein intake from pre-to post-intervention (+31%), while LDa had no difference. This increase resulted in the RDa group having a higher (*p* = 0.02) protein intake post-intervention compared to the LDa group (mean difference = +0.28). Based on our design, there were also group*intervention interactions showing that RDa increased intakes of micronutrients that are known to support bone growth in adolescents including vitamin D (+79%), calcium (+64%), phosphorous (+63%), and potassium (+42%) from pre-to post-intervention, while LDa had no changes in the intakes of these nutrients.

**FIGURE 1:**
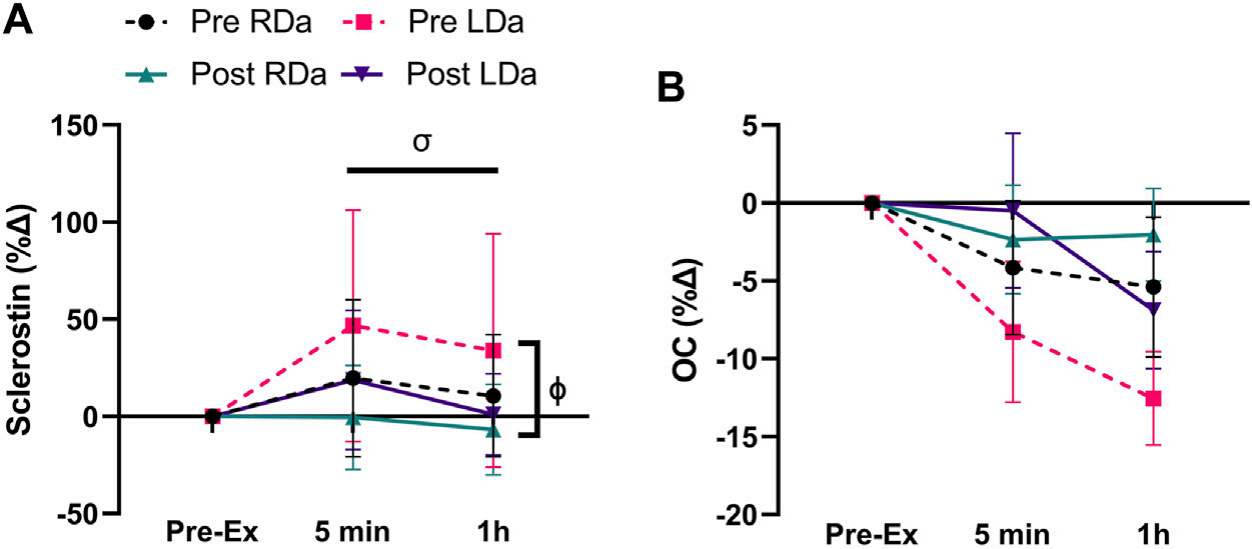
Serum sclerostin and osteocalcin (OC) percent change to acute exercise at pre- and post-intervention in both RDa and LDa groups: percent change of serum sclerostin **(A)** and OC **(B)** at 5 min and 1 h post-exercise relative to pre-exercise in both RDa (*n* = 14) and LDa (*n* = 16) groups. *σ* = main effect for time (*p* < 0.05) independent of intervention or group. *ϕ* = main effect for intervention (*p* < 0.05) independent of group. Data are reported as mean ± SD.

In terms of the biochemical markers (Table 2), there was an intervention*time interaction (*p* = 0.008), but no other significant interactions for sclerostin. Specifically, at pre-intervention, sclerostin increased from pre-exercise to 5 min (*p* = 0.004, +33.3 pg ml^−1^) and remained higher than its pre-exercise level at 1 h post-exercise (*p* = 0.02, +20.4 pg ml^−1^), while at post-intervention, there was a decrease in sclerostin from 5 min to 1 h post-exercise (*p* = 0.02, −19.2 pg ml^−1^) irrespective of group (Table 2). For sclerostin percent change, there was no significant main effect for group and no interactions. However, main effects were found for intervention (*p* = 0.03, −25%), and time (*p* = 0.04, +11% from pre to 5 min post-exercise), reflecting an overall decrease in the relative post-exercise sclerostin response pre-to post-intervention (Figure 1A).

**TABLE 2:** Serum concentrations of bone biochemical markers and osteokines at rest (pre-exercise) and in response to acute plyometric exercise at pre- and post-intervention in the higher dairy intake (RDa) and the low dairy intake (LDa) groups.

Protein	Group	Pre-intervention acute exercise response			Post-intervention acute exercise response			Significant interactions
		Pre	5 min	1 h	Pre	5 min	1 h	
Sclerostin (pg·ml^−1^)	RDa (*n* = 14)	153.8 ± 43.4	177.0 ± 58.1^$^	164.0 ± 43.8^$^	155.4 ± 31.5	152.7 ± 46.09^&^	140.8 ± 31.3^#^	intervention*time *p* = 0.008
	LDa (*n* = 16)	134.0 ± 49.6	175.4 ± 52.2^$^	160.7 ± 48.8^$^	151.0 ± 46.0	168.0 ± 52.3^&^	144.4 ± 35.3^#^	
Osteocalcin^b^ (ng·ml^−1^)	RDa (*n* = 14)	19.8 ± 8.0	18.5 ± 7.0	18.3 ± 7.3	20.6 ± 8.5	20.0 ± 8.2	20.2 ± 8.7	
	LDa (*n* = 16)	22.5 ± 12.2	19.9 ± 10.6	19.0 ± 9.2	22.2 ± 9.4	21.9 ± 9.5	20.5 ± 8.9	
OPG^b,c^ (pg·ml^−1^)	RDa (*n* = 14)	265.2 ± 84.5	271.6 ± 81.6	240.5 ± 53.4	281.6 ± 75.5	282.5 ± 64.7	272.3 ± 51.7	
	LDa (*n* = 16)	291.8 ± 105.9	284.7 ± 116.4	260.9 ± 79.6	272.6 ± 86.2	272.9 ± 88.1	272.6 ± 104.1	
RANKL^b.c^ (pg·ml^−1^)	RDa (*n* = 13)^§^	101.5 ± 147.9	116.6 ± 169.2	101.3 ± 149.1	113.9 ± 147.9	117.2 ± 157.6	96.0 ± 128.3	
	LDa (*n* = 16)	64.4 ± 57.3	61.3 ± 56.6	58.7 ± 59.9	63.4 ± 51.0	60.1 ± 44.4	51.7 ± 48.2	
OPG:RANKL (AU)	RDa (*n* = 13)^§^	5.0 ± 2.7	4.3 ± 2.2	4.6 ± 2.6	5.2 ± 5.5	4.9 ± 4.8	7.5 ± 9.5^$%^	intervention*time *p* = 0.03
	LDa (*n* = 16)	7.5 ± 5.4	8.1 ± 6.2	9.7 ± 8.7	7.1 ± 6.1	6.7 ± 4.9	10.0 ± 8.6^$%^	
βCTX^a,b,c^ (pg·ml^−1^	RDa (*n* = 14)	941.6 ± 221.8	1,026.2 ± 219.5	804.2 ± 233.3	821.0 ± 217.8	912.7 ± 252.6	728.1 ± 208.7	
	LDa (*n* = 16)	942.1 ± 391.2	963.6 ± 395.3	702.5 ± 370.1	964.0 ± 419.8	988.8 ± 361.0	762.1 ± 311.3	

Data are reported as mean ± SD. OPG, osteoprotegerin; RANKL, receptor activator nuclear factor kappa B ligand, βCTX, C-terminal telopeptide of type 1 collagen. § = No serum available for one participant to measure RANKL. A three-way RMANOVA, was used to examine main effects for group, time, intervention and their interactions. When a main effect of time is present, pairwise comparisons across timepoints are shown for groups and pre-/post-intervention values combined: a = *p*< 0.05 5 min post-exercise compared to pre-exercise, b = *p*< 0.05 1 h post-exercise compared to pre-exercise, c = *p*< 0.05 1 h post-exercise compared to 5 min post-exercise independent of intervention and group. When an Intervention*Time interaction is present, pairwise comparisons for both groups at either pre- or post-intervention: $ = *p*< 0.05 compared to pre-exercise, % = *p*< 0.05 compared to 5 min post-exercise, & = *p*< 0.05 compared to 1 h post-exercise. # = *p*< 0.05 difference at that timepoint compared to the same timepoint at pre-intervention.

For total OC, a main effect was found for time (*p* = 0.005) but no significant main effects for intervention or group, and no significant interactions. Thus, the time effect reflects a small but significant decrease from pre-to 1 h post-exercise (*p* = 0.001, −1719.9 pg ml^−1^) in both groups and pre/post-intervention combined (Table 2). OC percent change showed no significant main effects or interactions (Figure 1B).

**FIGURE 2:**
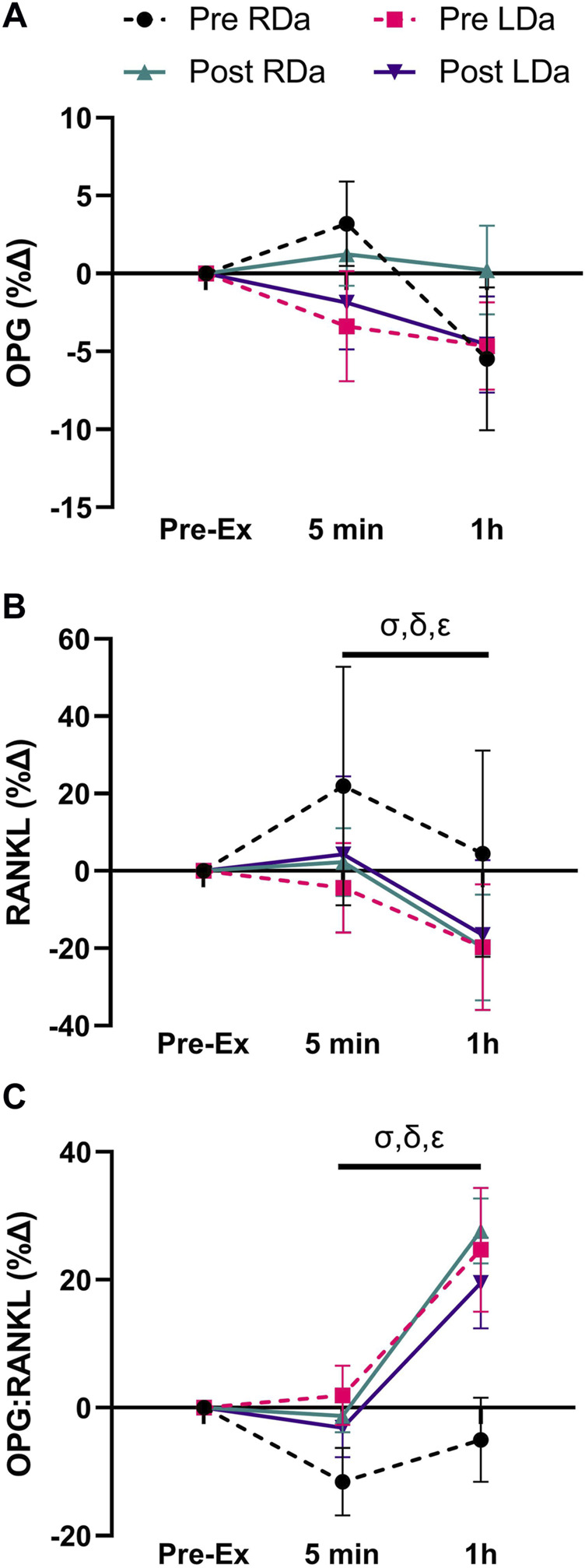
Serum osteoprotegerin (OPG), receptor activator nuclear factor kappa B ligand (RANKL), and OPG:RANKL ratio percent change to acute exercise at pre- and post-intervention in both RDa and LDa groups: percent change of serum OPG **(A)**, RANKL **(B)**, and OPG:RANKL ratio **(C)** at 5 min and 1 h post-exercise relative to pre-exercise in both RDa (*n* = 14, 13 and 13, respectively) and LDa (*n* = 15 for all markers) groups. *σ* = main effect for time (*p* < 0.05) independent of intervention or group. Following the intervention*group interaction, *δ* = pairwise comparison between RDa and LDa at pre-intervention (*p* < 0.05), *ε* = pairwise comparison between pre- and post-intervention in RDa only (*p* < 0.05). Data are reported as mean ± SD.

We found no significant main effects for intervention or group for OPG, and no significant interactions (Table 2). There was a main effect for time (*p* = 0.01), reflecting a decrease in OPG at the 1 h post-exercise timepoint compared to pre-exercise (*p* = 0.02, −16.2 pg ml^−1^) and 5 min post-exercise (*p* = 0.02, −16.4 pg ml^−1^) in both groups and pre-/post-intervention combined (Table 2). OPG percent change showed no significant main effects or interactions (Figure 2A).

RANKL showed a significant main time effect (*p* < 0.0001) but no intervention or group effects, and no significant interactions (Table 2). Pairwise comparisons for time identified no difference in RANKL between pre- and 5 min post-exercise, while it was lower at 1 h post-exercise compared to both pre-exercise (*p* < 0.001, −8.9 pg ml^−1^) and 5 min post-exercise (*p* < 0.001, −11.9 pg ml^−1^) in both groups and pre-/post-intervention combined (Table 2). However, RANKL percent change showed an intervention*group interaction (*p* = 0.005), which was the result of RDa having a relatively positive percent change following acute exercise compared to LDa at pre-intervention (*p* = 0.003, +25%). However, the relative responses of RDa and LDa at post-intervention were not different. Specifically, the RANKL percent change response to acute exercise had a net decrease from pre-to post-intervention in the RDa group (*p* = 0.003; −22%) whereas LDa’s percent change response to acute exercise was unchanged. There was also a main effect for time (*p* < 0.001, −19%), but with no other significant interactions (Figure 2B).

We found an intervention*time interaction (*p* = 0.03) for the OPG:RANKL ratio reflecting no changes in OPG:RANKL from pre to post-exercise at pre-intervention, while the ratio was higher 1 h post-exercise compared to pre-exercise (*p* < 0.001, +2.6 AU) and 5 min post-exercise (*p* < 0.001, +2.9 AU) post-intervention in both groups (Table 2). No other significant interactions were found. However, the OPG:RANKL ratio percent change showed a main effect for time (*p* < 0.001, +20%) and an intervention*group (*p* = 0.003) interaction, but no other significant interactions. Pairwise comparisons for the intervention*group interaction showed that at pre-intervention, the relative OPG:RANKL ratio was lower in the RDa compared to LDa group (*p* = 0.01, −22%) but was not different post-intervention, and that the relative acute response of the OPG:RANKL ratio to exercise increased post-intervention only in RDa (*p* = 0.001; +21%) whereas it did not change in LDa (Figure 2C).

**FIGURE 3:**
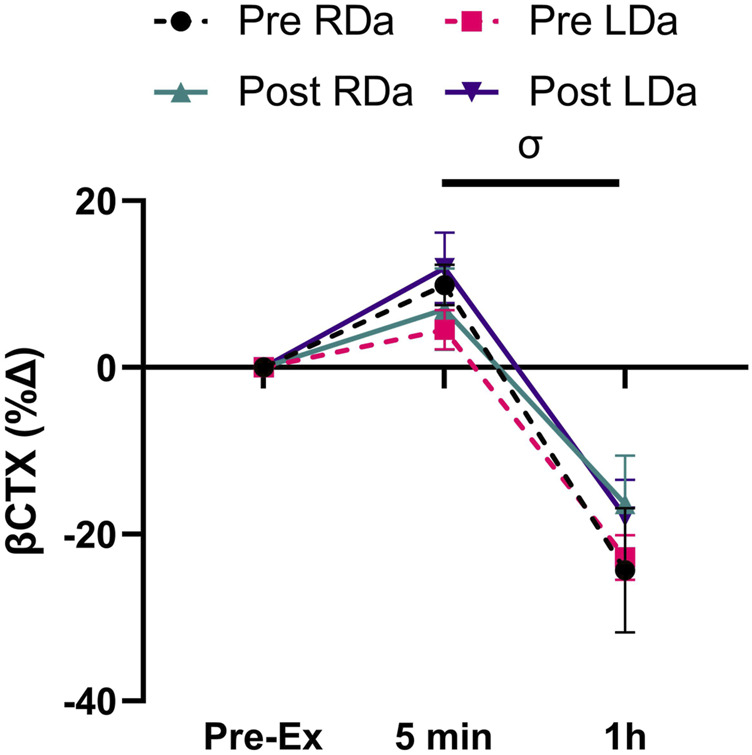
Serum C-terminal telopeptide of type 1 collagen (βCTX) percent change to acute exercise at pre- and post-intervention in both RDa and LDa groups: percent change of serum βCTX at 5 min and 1 h post-exercise relative to pre-exercise in both RDa (*n* = 14) and LDa (*n* = 16) groups. *σ* = main effect for time (*p* < 0.05) independent of intervention or group. Data are reported as mean ± SD.

Finally, βCTX concentration showed a main time effect (*p* < 0.001) but no significant main effects for intervention or group and no significant interactions (Table 2). Pairwise comparisons for time identified an increase from pre- to 5 min post-exercise (*p* = 0.001, +63.5 pg ml^−1^), while 1 h post-exercise βCTX was lower than both pre-exercise (*p* < 0.001, −187.1 pg ml^−1^) and 5 min post-exercise (*p* < 0.001, −250.6 pg ml^−1^) in both groups and pre-/post-intervention combined. For βCTX percent change, there was a main effect for time (*p* < 0.001, −28.4%) but no main effects for intervention or group and no significant interactions (Figure 3).

## 4 Discussion

This is the first study to compare the influence of a 12-week lifestyle modification (dietary counselling and exercise training) intervention on the response of bone biochemical markers and regulators to acute exercise, and to specifically isolate the effect of increased dairy intake during the intervention. The 12-week intervention, independent of dairy consumption, reduced the sclerostin response and augmented the OPG:RANKL ratio response to acute exercise. Dairy intake during the intervention influenced the relative RANKL and OPG:RANKL ratio responses to exercise in the RDa group, bringing this group’s RANKL response closer to the LDa post-exercise response, which was lower at pre-intervention. Aside from this, increased dairy consumption, and subsequently increased habitual intake of bone-supporting nutrients, during the intervention did not further influence the regulation of bone markers in response to acute exercise. Thus, our results suggest that a lifestyle intervention of healthy eating and exercise training for weight management in adolescents with OW/OB may not influence the short-term response of bone turnover markers (βCTX and OC) to acute exercise but may influence upstream regulators of bone turnover (i.e., osteokines including sclerostin and OPG:RANKL).

In this study, we showed that sclerostin increased immediately post-exercise and remained elevated at 1 h post-exercise at pre-intervention in adolescent females with OW/OB. The pre-intervention response was similar to our previous findings (Kurgan et al., 2020), which showed adolescent females with OW/OB had a transient post-exercise increase in sclerostin that is more characteristic of the adult bone response (Kouvelioti et al., 2019), while normal weight age-matched controls had no response in sclerostin to acute exercise. However, following 12 weeks of a lifestyle intervention, the transient post-exercise increase in sclerostin, which we initially observed in those with OW/OB, was blunted, independent of dairy intake. This new finding is important because sclerostin is known to inhibit bone formation (Poole et al., 2005), suggesting that during adolescence, when peak bone mass is accrued, the transient post-exercise increase in sclerostin is likely a maladaptation related to OW/OB. Thus, our 12-week intervention involving diet modification and exercise training, corrected this perturbation/maladaptation in our group of adolescent females with OW/OB. It has been previously shown *in vivo* (Robling et al., 2008) and *in vitro* (Spatz et al., 2015) that osteocyte sclerostin expression decreases with long-term mechanical loading. This finding is likely why other researchers have observed a negative association of circulating sclerostin with long-term exercise training in humans (Amrein et al., 2012) and potentially why we see a difference in the response to acute exercise in this study. Our lifestyle intervention also induced a decrease in fat mass (and an increase in lean mass) in both groups. Indeed, observational studies have found circulating sclerostin to be positively correlated with fat-free mass in adult females (Sheng et al., 2012). Additionally, while we found no response in whole-body measures of insulin resistance in our main IDEAL Study cohort [reported elsewhere (Skelly et al., 2021)], it is important to note that serum sclerostin levels appear to be elevated in individuals with prediabetes and correlate with insulin resistance in skeletal muscle, liver, and adipose tissue (Daniele et al., 2015). Additionally, in our previous assessment of adolescent females, we found those with OW/OB had a higher and more sustained increase of insulin following consumption of a high carbohydrate breakfast and acute plyometric exercise compared to those with normal weight and this difference mimicked the differential response of sclerostin between groups (Kurgan et al., 2020). Therefore, tissue-specific changes in metabolism/insulin sensitivity, not detected by our whole-body resting measures, throughout this study may also explain the changes we observed in sclerostin regulation to exercise. We propose that alterations in fat mass and the subsequent metabolic and biochemical changes that occur in tandem can govern sclerostin content within the bone microenvironment and influence the post-exercise response. Further studies are needed to identify the factors regulating the changes in the response of sclerostin to acute exercise and the biological significance of this adaptation (e.g., long-term influence on tissue growth and metabolism).

Given sclerostin is a critical regulator of osteoblast activity, we next examined OC, an enzymatic marker of bone turnover and osteoblast activity (Lee et al., 2007). OC has previously been shown to transiently decrease in response to acute exercise, suggesting that osteoblasts contribute less to the acute bone response to exercise (Dolan et al., 2020). Findings from this study provide evidence of no influence of a lifestyle modification intervention involving diet modification and exercise training on the regulation of OC following acute exercise. This finding was expected, as previous studies in normal-weight adults (Scott et al., 2010) and children (Pomerants et al., 2008; Theocharidis et al., 2020) rarely detect increases in bone formation (i.e., P1NP) following acute exercise (Dolan et al., 2020). In addition, this analysis only included a measure of total OC, and not the assessment of other proteoforms of OC (e.g., undercarboxylated) which are known to influence metabolism (Mizokami et al., 2013; Rehder et al., 2015; Tsuka et al., 2015; Gao et al., 2016; Guo et al., 2017; Mukai et al., 2021). Taken together, our study does not support the effect of a diet and exercise intervention influencing OC’s response to acute exercise in adolescent females with OW/OB.

The bone resorption marker, βCTX, increased immediately after acute exercise with a subsequent dip at 1 h post-exercise, which was not influenced by the intervention or level of dairy intake. This response of βCTX mimics data from adults (Dolan et al., 2020; Dolan et al., 2022) and contrasts our previous analysis that found a progressive reduction at 5 min and 1 h post-exercise in adolescents with either normal weight or OW/OB (Kurgan et al., 2020). Furthermore, we also did not find an effect of dairy consumption on the response of βCTX to acute exercise, despite RDa having increased habitual protein, vitamin D, calcium, phosphorous, and potassium intake while LDa had no change. This suggests that exercise training and increasing habitual intake of bone anabolic nutrients, at least in the shorter term (12-weeks) and in this cohort, does not influence the immediate response of βCTX to acute exercise.

Absolute concentrations of OPG and RANKL decreased slightly in response to acute exercise and this response was not influenced by either the intervention or dairy intake. However, our percent change data identified RANKL’s acute response to exercise adapted differently in the RDa and LDa groups, with only the RDa group having a lower relative RANKL response to acute exercise at post-intervention compared to pre-intervention indicating that dairy intake during the intervention led to a lesser bone resorptive response post-intervention in RDa. Likewise, we observed an effect of dairy intake on the percent change of OPG:RANKL, reflecting that the RDa group increased their relative anabolic response of this pathway from pre-to post-intervention. However, this interaction resulted from a pre-intervention difference in the percent change response between RDa and LDa. Thus, we acknowledge that it is also possible that the differential response between RDa and LDa to the intervention could be driven by the pre-intervention difference rather than the influence of dairy during the intervention.

Our study had several limitations. A significant limitation is that the control group was not included in the analysis (due to a very low number of control participants completing the two acute exercise bouts). Despite the short duration (12 weeks) of the intervention, the exclusion of the control group has implications for the current data as we are unable to account for the effect of general growth and maturation on our outcomes. There were also several statistical trends in main effects and interactions, including those for RANKL, OPG:RANKL, and βCTX (not described herein), which were likely a result of the small sample size. We also lack timepoints that extend past 1 h post-exercise. Indeed, some of these markers are transiently sensitive days following acute exercise (Sale et al., 2015), while we have previously shown that others return to baseline levels around 1 h post-exercise (Falk et al., 2016; Dekker et al., 2017; Kouvelioti et al., 2019; Kurgan et al., 2020). While we endeavoured to include a 24 h timepoint, ∼30% of participants did not return for sampling at 24 h resulting in too many missed data points for this timepoint to be included in our analysis. An intrinsic limitation of this study (and other similar studies measuring osteokines in human blood) is that we cannot decipher the extent to which sclerostin, OPG and RANKL measured in serum reflect bone tissue protein levels (it is not possible or feasible to obtain bone biopsies). Lastly, due to specific concerns in this population, we provided them with a small, consistent breakfast high in carbohydrates before their acute exercise session, which is known to blunt bone turnover (Sale et al., 2015; Townsend et al., 2017). This may have reduced some of the acute responses and prevented the identification of key differences (Sale et al., 2015; Townsend et al., 2017). It is important to note that we have used this same pre-exercise breakfast across studies within our lab (Falk et al., 2016; Dekker et al., 2017; Kouvelioti et al., 2019; Kurgan et al., 2020) to allow for appropriate comparison between studies. While there are several limitations, this study provides unique insight into bone metabolism by examining the response of bone biochemical markers to acute exercise both pre- and post-intervention in a group of adolescent females with OW/OB. This unique, complex, study design led to the identification of proteins that adapted to the acute stress of exercise following our lifestyle modification intervention that the assessment of resting values may not otherwise identify (Josse et al., 2020). Future studies should assess the response of bone turnover biomarkers and upstream osteokine regulators to acute exercise pre- and post-exercise training across different age groups, levels of adiposity, levels of fitness (e.g., sedentary to high-performance athletes), and in disease/clinical populations (e.g., metabolic syndrome) to provide further context to our findings.

## 5 Conclusion

This study utilizes a unique design to add to a body of literature examining the effects of exercise and nutrition (specifically, dairy intake) on bone remodelling and metabolism in adolescent females with OW/OB. We found that our lifestyle modification intervention of healthy dietary counselling and exercise training (3×/week) blunted the increase in sclerostin and augmented the increase in OPG:RANKL following acute exercise, while dairy product consumption did not further influence these responses. To our knowledge, there is no previous study designed to investigate the influence of a lifestyle modification intervention for weight management with higher dairy intake vs. low dairy intake on the acute bone metabolic response to exercise, particularly in this demographic. These findings are critical to furthering our understanding of the influence of OW/OB on bone metabolism in adolescent females, which dietary factors influence bone metabolism and the mechanisms that lead to improved bone growth and achievement of peak bone mass with multiple bouts of acute exercise (i.e., exercise training).

## Data Availability

The raw data supporting the findings of this study are available from the corresponding author ARJ, upon reasonable request.
